# Bulbar Artery Injury With Bladder Hematoma and Severe Anemia Due to Traumatic Foley Catheter Removal

**DOI:** 10.7759/cureus.33488

**Published:** 2023-01-07

**Authors:** Dev P Patel, Guillermo Izquierdo-Pretel

**Affiliations:** 1 Internal Medicine, Herbert Wertheim College of Medicine at Florida International University, Miami, USA

**Keywords:** blood loss anemia, substance abuse, urological emergency, bladder trauma, bladder hematoma, foley catheter injury

## Abstract

This case is described with the aim of informing about the high-level suspicion of bladder or urethral injuries in patients with traumatic Foley removal and their prolonged bleeding that should alert clinicians for a prompt urological intervention. A patient was initially admitted to the ICU with delirium and organ dysfunction due to an overdose of drugs. On the second day of his admission, he unintentionally removed his Foley catheter, which led to a course of gross hematuria. He was managed conservatively. After three weeks of hospitalization and stabilization, his profuse, constant bleeding was finally addressed. CT and ultrasound imaging was performed and revealed that his bladder was at an abnormal size and filled with blood. A cystoscopy and a fulguration of the bulbar artery were completed. Quick relief and recovery were noted after the procedure was finalized.

## Introduction

Forced removal of a Foley catheter is common amongst agitated or delirious patients. Removing a Foley by such means is often traumatic to the urethra in males and often leads to urethral injury. However, the presentation of a bladder hematoma is a much more serious consequence of unintentional Foley catheter removal and needs immediate surveillance and surgical intervention. Therefore, clinicians must maintain a high level of suspicion for catheter-related bladder injuries, especially in a patient with altered mental status, prolonged hematuria, and rapidly dropping hemoglobin levels.

## Case presentation

A 30-year-old male patient with a past medical history of bipolar disorder and polysubstance drug abuse presented to a tertiary hospital in Miami with altered mental status due to polysubstance overdose. Workup revealed acute kidney failure, rhabdomyolysis, hepatic failure, lactic acidosis, and severe electrolyte imbalances (Table 1). Upon arrival to the ED, he was unresponsive, apneic, showed agonal breathing, and had fixed and unequal pupils. There was suspected benzodiazepine, cocaine, fentanyl, and alcohol use. The patient was quickly intubated and carefully monitored. However, during an incident of agitation overnight, the patient forcefully removed his Foley catheter with the balloon still inflated and began experiencing hematuria. The patient required a urology consult. The patient was voiding clear yellow urine prior to Foley removal. Ultrasound of the bladder showed decompression with some clots, and Urology placed another Foley with traction. Urology initially believed that the source of the bleeding was most likely from the prostatic urethra rather than the bladder. The patient was extubated on day six and transferred to the hospitalist team. However, oliguria with dark, bloody urine and clots was still present. The patient was admitted due to severe suprapubic pain. His hospital stay continued to be complicated, as he required regular dialysis and was on treatment for Vancomycin-resistant Enterococcus bacteremia.

**Table 1:** Laboratory results at admission time. mmHg: Millimeters of mercury; mmol/L: Millimoles per litre; mcL: Microliter; g: Grams; mg: Milligrams; dL: Deciliter; L: Liter.

Laboratory	Admission time results	Reference values
Arterial pH	7.28	7.35-7.45
Arterial carbon dioxide (PCO2)	39 mmHg	35-45 mmHg
Oxygen arterial pressure (PO2)	211 mmHg	75-100 mmHg
Arterial bicarbonate (HCO3)	9 mmol/L	19-24 mmol/L
Arterial oxygen saturation	99 %	Over 92%
WBC count	9.9 x10^(3)/mcL	4.0-10.5 x 10^(3)/mcL
Hemoglobin	13.4 g/dL	13.3-16.3 g/dL
Whole blood glucose	33 mg/dL	74-106 mg/dL
Whole Blood Sodium	140 mmol/L	137-145 mmol/L
Whole blood potassium	2.9 mmol/L	3.6-5.0 mmol/L
BUN level	49 mg/dL	9-20 mg/dL
Creatinine level	3.8 mg/dL	0.52-1.04 mg/dL
Calcium level	5.7 mg/dL	8.4-10.2 mg/dL
Total protein	5.3 g/dL	6.3-8.2 g/dL
Albumin level	2.6 g/dL	3.9-5.0 g/dL
Aspartate aminotransferase (AST)	7,500 unit/L	15-46 unit/L
Alanine transaminase (ALT)	3,979 unit/L	21-72 unit/L
Total bilirubin	4.4 mg/ dL	Less than 0.3 mg/dL
Lactic acid	5.4 mmol/L	0.5-2.0 mmol/L

**Figure 1:**
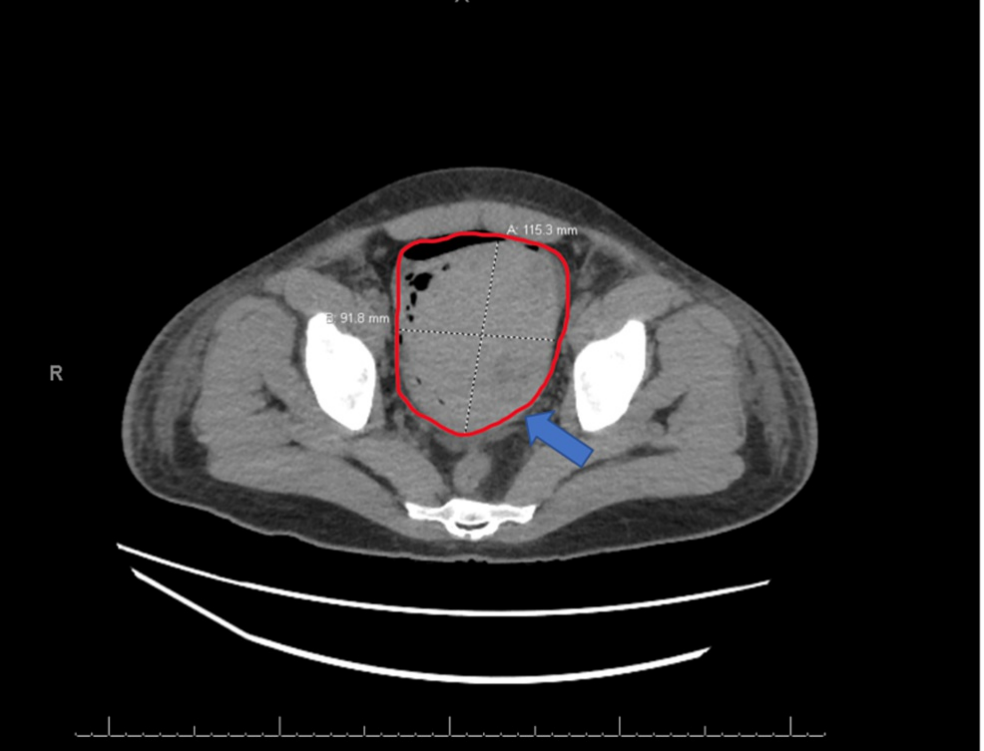
CT abdomen and pelvis with contrast: 12-cm hyperdense lesion in the urinary bladder, likely representing hematoma (blue arrow pointing at the hematoma circled in red). mm: Millimeters.

**Figure 2:**
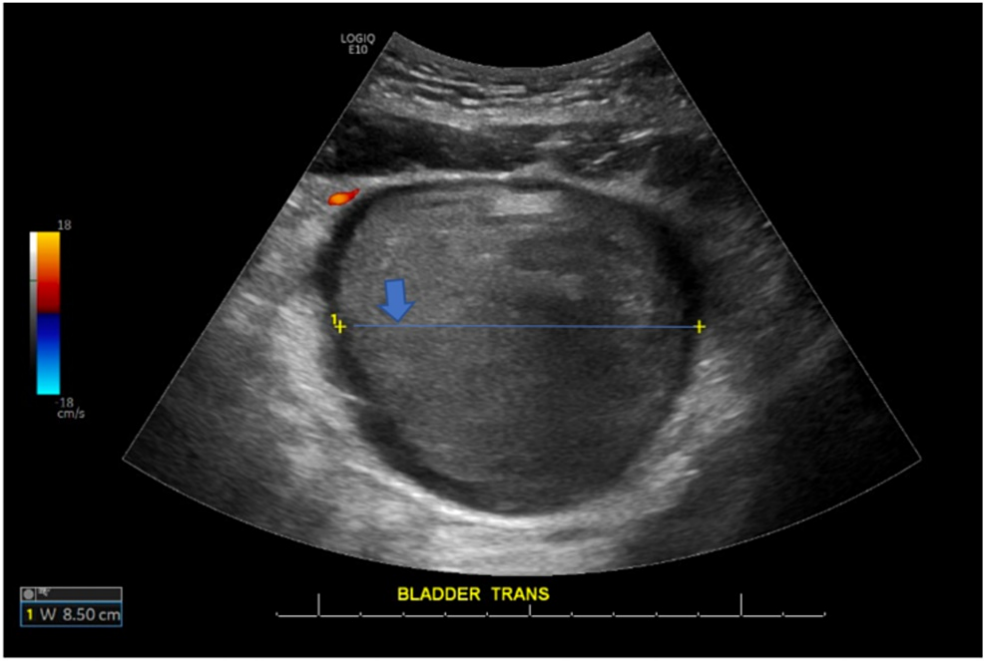
Ultrasound urinary bladder #1: 12.6 x 9.2 x 8.5 cm heterogeneous structure within the urinary bladder likely represent evolving hematoma (blue arrow pointing at the line delineating the width of the hematoma). W: Width; cm: Centimeters.

On day 20, the patient received a CT of the abdomen and pelvis with contrast, which showed a 12 cm hyperdense lesion in the urinary bladder, representing a hematoma (Figure 1). In addition, an ultrasound of the urinary bladder on the same day showed a 12.6 x 9.2 x 8.5 cm heterogeneous structure within the urinary bladder that represented an evolving hematoma (Figure 2). At this time, the patient's hemoglobin read 7.0 g/dL, while his initial hemoglobin levels at admission read 13.4 g/dL.

**Figure 3:**
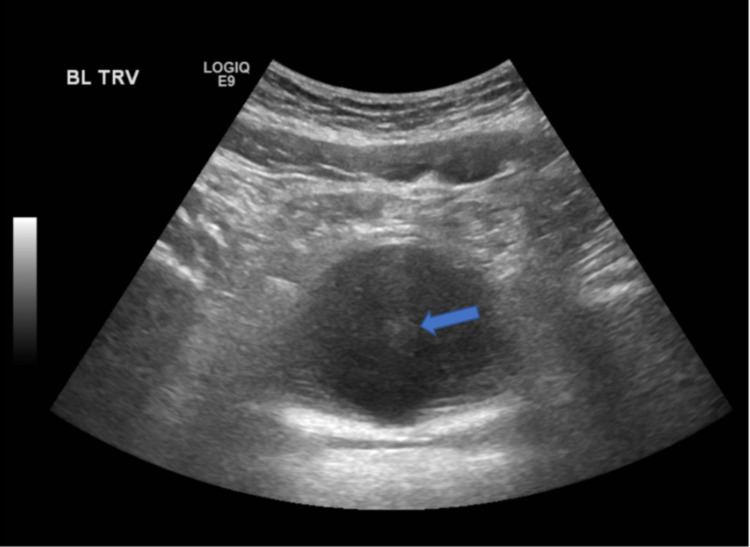
Post-op ultrasound of the pelvis: Ultrasound pelvis showed an under-distended urinary bladder with Foley catheter (blue arrow) in place and the previously seen large hematoma is no longer visualized.

Urology conducted a cystoscopy with clot evaluation and fulguration of bleeders on day 21. The bulbar artery was the source of the lesion. In terms of the procedure, a resectoscope sheath with the visual obturator and 30-degree lens was inserted atraumatically into the urethra. Using the Toomey syringe and Ellik evacuator, multiple clot fragments were irrigated until visualization improved. Then, a visual obturator was exchanged for the working element with the bipolar resection loop. The bladder mucosa was friable, and areas of bleeding from the posterior bladder wall were fulgurated. Post-op ultrasound of the pelvis showed an under-distended urinary bladder with a Foley catheter in place, and the previously seen large hematoma was no longer visualized (Figure 3). However, post-op lab work showed that his hemoglobin precipitously dropped to alarmingly low levels (5.1 g/dL).

Packed RBC transfusions and, eventually, five days of iron sucrose treatments were used to restore hemoglobin to acceptable values. As a result, the patient experienced rapid relief of symptoms post-surgery and was no longer bleeding out of his Foley catheter. In addition, his hemoglobin levels had finally stabilized (10.5 g/dL) one month after his initial trauma.

## Discussion

Suspected bladder trauma is usually the most common cause of bladder hematoma. They can be further subdivided into extraperitoneal and intraperitoneal injuries. The former generally occurs with pelvic fractures and can be managed with an indwelling catheter, while the latter usually occurs with high-energy impacts to an overdistended bladder and requires emergent surgical intervention [1,2]. Car crashes (47.3% of blunt mechanisms), falls from high places, heavy objects falling on the lower abdomen, gunshots (87.3% of penetrating mechanisms), knives, shrapnel, and improvised explosive devices (IEDs) have all been linked to bladder trauma. Blunt trauma accounts for approximately 60-85%, and penetrating trauma accounts for 15-40% [3]. A distended bladder has a higher risk of rupture than an empty one, as the bladder gets anatomically displaced from its protective location in the pelvis [3].

Mortality rates for bladder injury range from 0 to 34%, with an average rate of 8% [1,2,3]. Some complications due to the trauma or surgical intervention can include urinary incontinence, wound dehiscence, decreased bladder capacity from over-debridement, persistent urinary extravasation, pelvic abscess from an infected hematoma, intraabdominal infection, fistula, UTI, and urinary urgency [1].

The differential diagnosis for frank hematuria in male patients is wide and include testicular trauma, penile trauma, urethral trauma, pelvic fractures, ureteral trauma, carcinoma of the bladder, infectious hemorrhagic cystitis, bladder trauma, urinary infection, benign prostate hypertrophy, and several other less common reasons [4]. In our patient, there was an injury to the bulbar artery, leading to a high-pressure bleed and buildup of blood in the bladder. As the bulbar artery is near the most proximal portion of the penis and distal to the bladder, the clogging of the Foley catheter leads to retrograde blood flow into the bladder. Common signs and symptoms include suprapubic tenderness, low urine output, painful urination, fever, back pain, and feeling of bladder fullness and urinary urgency [2].

Lab work such as complete blood count, basic metabolic panel, and urine analysis should be obtained as part of the workup. In hemodynamically stable patients, CT abdomen and pelvis or ultrasound bladder can diagnose bladder hematomas. However, in instances of hemodynamic instability, cystoscopy is indicated because the source of the bleeding must be tended to immediately [2]. With this patient, he was hemodynamically unstable for an extended period; however, the CT and ultrasound were the two modalities used to diagnose his hematoma. While the gold standard remains cystography (sensitivity and specificity of 95% and 100%, respectively), ultrasound is a quick and reliable imaging modality in emergency cases [5]. Current data has not explored the efficacy of point-of-care ultrasound (POCUS) versus conventional ultrasound in diagnosing bladder injury. It may be worth investigating whether POCUS could help quickly diagnose these injuries [5].

Emergent surgical intervention includes cystoscopy and fulguration of bleeding vessels. Interestingly, the bulbar artery was found to be the culprit of the hematoma in this case, leading to a drop in hemoglobin from 13.4 on day 1 to 5.1 on day 22. The patient's rapid deterioration and anemic presentation alerted the hospitalist team to look for additional interventions, as the frequent packed RBC transfusions and iron sucrose treatments were not stabilizing the hemoglobin levels appropriately. Compression of the hemorrhaging site is known as the traditional and commonly used method to control bleeding. Although this traditional method was used with the patient, it was unsuccessful. A gradual inflation method has also been recommended in the literature for intraurethral bleeding, but it was not used in our case [6].

Prevention of traumatic Foley catheter removals and early identification of malpositioning can certainly reduce long-term complications related to bladder injury, decrease a patient's hospital length of stay, increase patient satisfaction, decrease catheter-associated UTIs, and improve hospital quality scores [7]. It requires an interprofessional team approach to identify at-risk patients and create an environment that reduces the likelihood of this traumatic event. Namely, patients with delirium or dementia are at high risk for intentional or unintentional traumatic Foley catheter removal. In the case we described, the patient had a significantly altered mental state when he pulled out his catheter. Hospital personnel should monitor suspiciously mispositioned catheters and use standard preventative measures such as taping and covering the catheter underneath the thigh to obscure it from the patient's sight and prevent them from reaching it [7]. Additional measures include using a diaper or mesh underwear with pads, decoy Foleys, mitts, and in extreme cases, restraints, sedation, or constant monitoring with a sitter for the highest-risk patients [7]. Each individual needs to be evaluated case by case to determine the best prevention method. Finally, in the case of intentional or unintentional removal, it is important to check the Foley balloon for any missing pieces or fragments because these pieces can remain in the bladder and might require removal via cystoscopy to prevent calcification and stone formation. Literature does not show evidence that using a larger (30 mL) balloon Foley minimizes unintentional injury. Although the balloon is larger and more resistant to being pulled out while inflated, it would lead to more damage to the urethra if a patient were to pull the Foley out [7]. Furthermore, larger catheter balloons may lead to increased irritation and patient stimulation, making the patient more prone to self-injury. Thus, the use of a larger balloon Foley is controversial. Amid many other complications, bladder injury and trauma due to unintentional Foley catheter removal may often be overlooked. There should be a low threshold for urological consultation and a high degree of clinical suspicion if a patient is actively bleeding for an extensive period with rapidly lowering hemoglobin levels, requiring multiple packed RBC transfusions.

## Conclusions

It is encouraged to maintain a high level of clinical suspicion for bladder injuries in patients with traumatic Foley removal and their prolonged bleeding. This should alert clinicians for a prompt urological intervention, especially in patients with altered mental status, prolonged hematuria, and rapidly dropping hemoglobin levels.
